# Etelcalcetide, A Novel Calcimimetic, Prevents Vascular Calcification in A Rat Model of Renal Insufficiency with Secondary Hyperparathyroidism

**DOI:** 10.1007/s00223-017-0319-7

**Published:** 2017-10-16

**Authors:** Longchuan Yu, James E. Tomlinson, Shawn T. Alexander, Kelly Hensley, Chun-Ya Han, Denise Dwyer, Marina Stolina, Charles Dean, William G. Goodman, William G. Richards, Xiaodong Li

**Affiliations:** Departments of Cardiometabolic Disorders and Comparative Biology and Safety Sciences, Amgen Inc., One Amgen Center Drive, MS: 36-2-A, Thousand Oaks, CA 91320 USA

**Keywords:** Etelcalcetide, Fibroblast growth factor-23 (FGF23), Parathyroid hormone, Secondary hyperparathyroidism, Vascular calcification

## Abstract

Etelcalcetide, a novel peptide agonist of the calcium-sensing receptor, prevents vascular calcification in a rat model of renal insufficiency with secondary hyperparathyroidism. Vascular calcification occurs frequently in patients with chronic kidney disease (CKD) and is a consequence of impaired mineral homeostasis and secondary hyperparathyroidism (SHPT). Etelcalcetide substantially lowers parathyroid hormone (PTH) and fibroblast growth factor-23 (FGF23) levels in SHPT patients on hemodialysis. This study compared the effects of etelcalcetide and paricalcitol on vascular calcification in rats with adenine-induced CKD and SHPT. Uremia and SHPT were induced in male Wistar rats fed a diet supplemented with 0.75% adenine for 4 weeks. Rats were injected with vehicle, etelcalcetide, or paricalcitol for 4 weeks from the beginning of adenine diet. Rats fed an adenine-free diet were included as nonuremic controls. Similar reductions in plasma PTH and parathyroid chief cell proliferation were observed in both etelcalcetide- and paricalcitol-treated rats. Serum calcium and phosphorus were significantly lower in etelcalcetide-treated uremic rats and was unchanged in paricalcitol-treated rats. Both serum FGF23 and aortic calcium content were significantly lower in etelcalcetide-treated uremic rats compared with either vehicle- or paricalcitol-treated uremic rats. The degree of aortic calcium content for etelcalcetide-treated rats was similar to that in nonuremic controls and corroborated findings of lack of histologic aortic mineralization in those groups. In conclusion, etelcalcetide and paricalcitol similarly attenuated progression of SHPT in an adenine rat model of CKD. However, etelcalcetide differentially prevented vascular calcification, at least in part, due to reductions in serum FGF23, calcium, and phosphorus levels.

## Introduction

Increased cardiovascular-related mortality is commonly observed in chronic kidney disease (CKD). The increased burden of cardiovascular disease is, in part, due to the development of vascular calcification [1]. Elevated levels of parathyroid hormone (PTH) and phosphorus in CKD patients are critical for the initiation and progression of vascular calcification in CKD [2]. While increased serum calcium is not common in untreated CKD patients with secondary hyperparathyroidism (SHPT), it may be elevated by certain treatment regimens further contributing to the development of vascular calcification.

Elevated levels of PTH and phosphorus in CKD patients may be managed by existing therapies. However, managing adverse cardiovascular consequences associated with SHPT is a major challenge and unmet medical need. Toward this end, a prospective, randomized clinical study showed that cinacalcet (Amgen Inc., Thousand Oaks, CA), a small molecule agonist of the calcium-sensing receptor (CaSR), plus low-dose vitamin D sterols may attenuate vascular and cardiac valve calcification by lowering circulating PTH, calcium, and phosphorus [3, 4]. Furthermore, a post hoc analysis of the Evaluation of Cinacalcet Hydrochloride to Lower Cardiovascular Events (EVOLVE) trial showed that cinacalcet significantly lowered serum fibroblast growth factor-23 (FGF23), and treatment-induced reductions in serum FGF23 were associated with lower rates of cardiovascular death and major cardiovascular events [5]. These results suggest the potential of calcimimetics to attenuate vascular calcification and cardiovascular mortality associated with SHPT in patients with CKD. In contrast, long-term administration of vitamin D sterols and calcium-containing phosphate binders to children and young adults with CKD was found to induce vascular calcification [6, 7]. In addition, paricalcitol (a vitamin D analog) significantly increased serum FGF23 in hemodialysis patients with SHPT [8, 9]. Moreover, randomized clinical studies demonstrated that hypercalcemia and hyperphosphatemia occurred more often in vitamin D analogs compared to cinacalcet [10]. To our knowledge, there are no prospective randomized clinical studies that describe the effects of the new generation of vitamin D analogs (e.g., paricalcitol) in comparison with placebo on vascular calcification.

Consistent with clinical studies, cinacalcet or other calcimimetics effectively decrease PTH levels and prevent vascular calcification in animal models of SHPT and vascular calcification [11–14]. In comparison, inconsistent results are reported for the effects of paricalcitol on vascular calcification in preclinical studies, whereby some studies have shown no effect on vascular calcification in rat models of SHPT [15–17]. It is important to note that vehicle-treated uremic rats did not develop vascular calcification in those studies. Other reports show that paricalcitol slightly aggravated vascular calcification in uremic rats [18, 19]. Two reports show that paricalcitol reduced vascular calcification in mice [20, 21]. The reasons for the inconsistent findings may be related to the experimental model or the dose used.

Etelcalcetide (formerly AMG 416; Amgen Inc., Thousand Oaks, CA) is a novel, selective peptide agonist of the CaSR that lowers circulating PTH levels in animal models of SHPT [22, 23] and in clinical studies of patients with SHPT on hemodialysis [24, 25]. It also lowers circulating FGF23 levels, calcium, and phosphorus in multiple clinical studies. Unlike cinacalcet, etelcalcetide is administered intravenously thrice weekly at the end of each hemodialysis session, which may improve the compliance and adherence of calcimimetic therapy. It is important to understand the effect of etelcalcetide on vascular calcification in preclinical models. In addition, to better clarify the effects of paricalcitol, an approved treatment option for SHPT, on vascular calcification, we compared side by side the effects of low-dose paricalcitol and etelcalcetide on vascular calcification under the same experimental conditions (rat model of SHPT induced by dietary adenine).

## Materials and Methods

### Animals and Dosing

Twelve-week-old male Wistar rats (*N* = 96; average weight of ~406 g) obtained from Charles River Labs (Raleigh, NC) were fed a low-protein (2.5%) and high-phosphate (0.92%) diet (Teklad Custom Diet, TD130126, Harlan Laboratories, Indianapolis, IN) for 1 week. Thereafter, 24 rats were maintained on the same diet to serve as nonuremic controls, and 72 rats were switched to the same base diet supplemented with 0.75% adenine (TD130127) for an additional 4 weeks to induce uremia and SHPT. Orally administered adenine is metabolized to 2,8-dihydroxyadenine, which precipitates and forms tubular crystals that injure the renal tissue. These 72 rats received etelcalcetide, vehicle, or paricalcitol treatment (*n* = 24 per group) within 24 h of adenine introduction. Etelcalcetide was formulated in vehicle (10 mM succinic acid, 0.85% NaCl, 0.9% benzyl alcohol, pH 4.5) and injected subcutaneously (SC) at 0.3 mg/kg once a day for 4 weeks. The SC route of administration was selected because of the length of the study and challenges associated with repeated i.v. dosing in animal studies. Subcutaneous administration of etelcalcetide has been shown to effectively decrease PTH and provides good drug exposure in animal studies [22]. Paricalcitol was injected intraperitoneally (IP) at 42 ng/kg three times per week for 4 weeks. The paricalcitol dose (42 ng/kg) was selected based on its nonsignificant effects on serum calcium while reducing PTH compared to higher doses (>42 ng/kg) [15]. The goal was to avoid the contribution of hypercalcemia induced by higher doses of paricalcitol to the development of vascular calcification. Twenty-four rats on adenine diet and 24 rats on adenine-free diet received vehicle by subcutaneous injection once a day for 4 weeks. Blood samples were collected through tail vein under isoflurane anesthesia before switching to adenine diet (predose) and prior to dosing at 2 and 4 weeks after the treatment. Rats were cared for in accordance with the *Guide for the Care and Use of Laboratory Animals, 8th* Edition [26].

### Serum/Plasma Analysis

Plasma PTH levels were quantified using rat (1–84) bioactive intact PTH ELISA kits (Immunotopics International, San Clemente, CA). Serum FGF23 levels were determined using rat PTH-FGF23 magnetic bead assay (Millipore, Billerica, MA), and PTH was also measured in the same assay. Serum creatinine was determined using the creatinine enzymatic reagent set (Pointe Scientific Inc., Canton, MI). Serum blood urea nitrogen (BUN), total calcium, and phosphorus concentrations were determined using a blood chemistry analyzer (Olympus AU 400, Olympus America Inc., Melville, NY).

### Kidney Histology

Kidneys were fixed in 10% neutral buffered formalin (NBF), embedded in paraffin, and sectioned at 4 µm thickness and stained with hematoxylin and eosin. Kidney sections were also stained with Masson’s Trichrome for collagen. Kidney tubulointerstitial damage was evaluated semiquantitatively under light microscope, in which the grading of 0–5 was performed according to the extent of damaged tubulointerstitial area in the renal cortex as in previous investigations [27]: 0, normal; grade 1, <10%; grade 2, 10–25%; grade 3, 25–50%; grade 4, 50–75%; and grade 5, 75–100%.

### Parathyroid Gland Weights

The two parathyroid glands from each animal were carefully dissected and freed of surrounding tissue under a dissecting microscope. The weight of each pair of parathyroid glands was measured using the analytical grade scale (Mettler Toledo, Excellence Plus, XP205 DeltaRange^®^, *d* = 0.01 mg/0.1 mg). Twenty-one rats with no or only one isolated parathyroid gland were not included in the analysis of parathyroid gland weight.

### Ki-67 Immunohistochemistry (IHC) and Quantification

To measure proliferative indices in parathyroid gland, modified immunohistochemical-adapted staining methods for Ki-67 [28] and subsequent quantification were performed. In brief, paraffin-embedded parathyroid gland blocks were sectioned at 4 µm thickness and processed for IHC. The IHC staining of Ki-67 was performed with the Dako Autostainer Universal staining system (Dako Norden A/S, Glostrup, Denmark), using a mouse anti-rat Ki-67 antibody (Clone MIB-5; dilution 1:50; Dako Norden A/S, Glostrup, Denmark). A biotinylated rat anti-mouse secondary antibody (dilution 1:750; Jackson ImmunoResearch Laboratories, West Grove, PA) was applied, followed by streptavidin–horseradish–peroxidase (dilution 1:1500; PerkinElmer, Waltham, MA) and DAB plus (Dako Nordern A/S, Glostrup, Denmark). Whole images were scanned with an Aperio ScanScope^®^XT (Aperio, Vista, CA). Ki-67 immunoreactivity was analyzed using Visiomorph image analysis program (Visiompharm, Hørsholm, Denmark). Ki-67-stained parathyroid chief cells were counted for each sample. Parathyroid proliferative index was calculated and expressed as Ki-67-positive cell counts per area. Twenty-four rats with no or only one isolated parathyroid gland were not included in the analysis of Ki-67.

### Calcium and Phosphorus Content in Aortic Tissues

A piece of 10% NBF-fixed descending aortic tissue was dissected from the location of 2 cm distant to the top of aortic arch to the abdominal region (2–4 cm in length). The aortic tissue was weighed and then put into 0.15 N HCl at a volume/wet weight ratio of 1.5 mL per 100 mg tissue and held at room temperature for 24 h. Calcium and phosphorus concentrations were quantified from supernatants of digested tissue using an Olympus AU400 Clinical Chemistry Analyzer (Olympus Inc.). Calcium and phosphorus content was expressed as µg/g tissue weight [29]. Aortic samples were not successfully collected from 2 out of 96 rats and these 2 rats were not included in the analysis.

### Aortic Histology

Aortic arch and 1 cm of descending aortic tissue were collected and fixed in 10% neutral buffered formalin (NBF). Paraffin-embedded longitudinal rat aorta sections (4-μm thick) were prepared using standard histology procedures. The sections were subjected to von Kossa staining for assessing calcified lesions. Semiquantitative histologic scoring of aortic wall mineralization: Grade 1/minimal = focal patchy vascular mineralization of tunica media detectible, Grade 2/mild = up to 10% of total specimen mineralized, Grade 3/moderate = 10–50% of total aortic specimen mineralized, Grade 4/Marked >50% of total specimen mineralized.

### Statistical Analysis

All data are reported as means with standard error of the mean. Two-way ANOVA, followed by Tukey’s multiple comparison test was used to determine the differences between groups for serum creatinine, BUN, plasma PTH, FGF23, calcium, phosphorus, and body weight. One-way ANOVA followed by the Tukey’s multiple comparison test was used for analyses of histology score of kidney tubulointerstitial injury, parathyroid gland weight/body weight, and parathyroid proliferative index. Nonparametric Kruskal–Wallis test was used to compare calcium and phosphorus content in aortic tissues. Statistical analyses were performed using GraphPad Prism (version 6.02, GraphPad Software, Inc.; San Diego, CA, USA). *P* < 0.05 was used to identify significant differences between groups.

## Results

### Body Weight

The average body weights of all four groups were similar before the treatment (Table 1). During the 4-week experiment, there was a minor increase in body weight in the rats fed with adenine-free diet. The average body weights decreased markedly starting from week 1 in the rats receiving the adenine diet. There was no significant difference in body weight between the vehicle- and etelcalcetide- or paricalcitol-treated rats at weeks 1, 2, 3, and 4.

**Table 1:** Body weight

Time points	Adenine-free (*n* = 24)	Adenine diet		
		Vehicle (*n* = 24)	Etelcalcetide (*n* = 24)	Paricalcitol (*n* = 24)
Week 0	408 ± 5	400 ± 4	402 ± 4	400 ± 4
Week 1	399 ± 7	338 ± 3*	343 ± 4*	345 ± 4*
Week 2	418 ± 7	321 ± 4*	322 ± 4*	325 ± 4*
Week 3	421 ± 7	313 ± 4*	310 ± 4*	312 ± 4*
Week 4	425 ± 7	301 ± 3*	306 ± 5*	305 ± 3*

Values are mean ± SEM. SEM, standard error of the mean

*P < 0.05 versus the rats on adenine-free diet

### Serum Creatinine and BUN

**Fig. 1:**
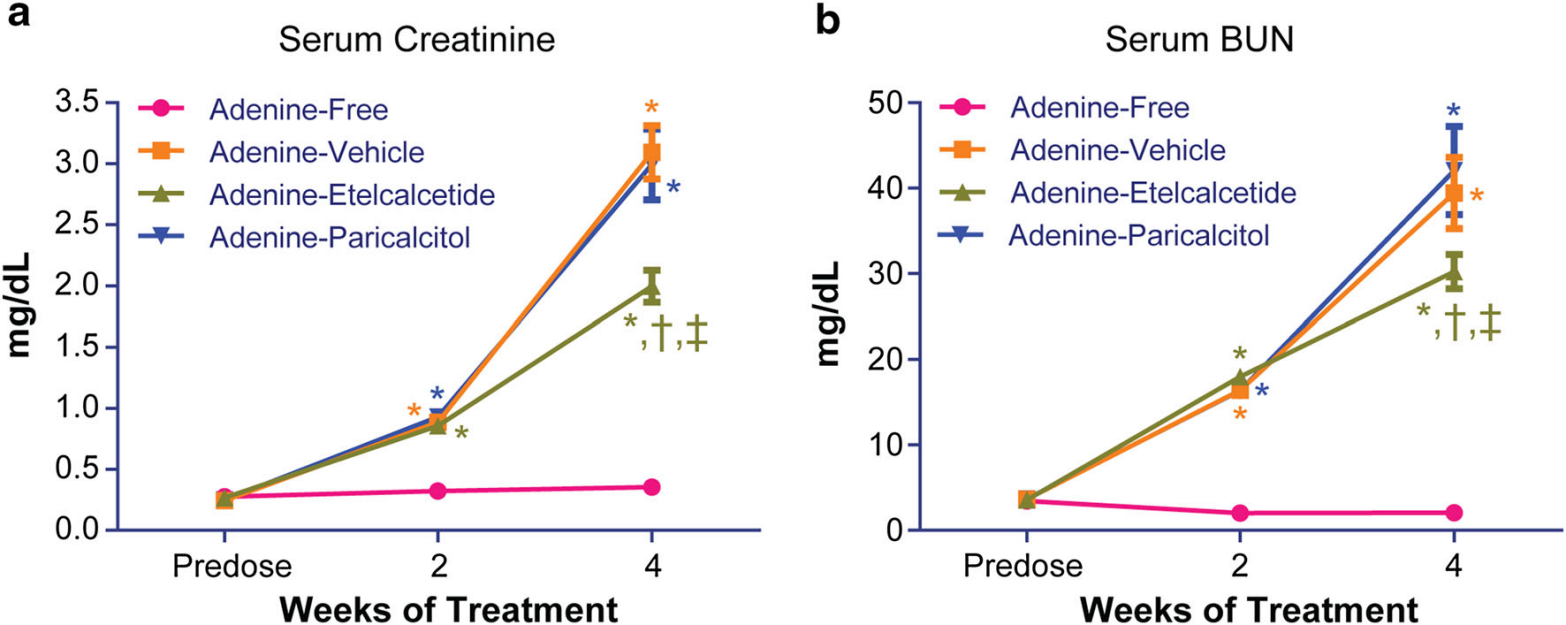
Serum creatinine and blood urea nitrogen (BUN) before and after treatment in rats on adenine or adenine-free diet. **a** Serum creatinine. **b** Serum BUN. Data are expressed as mean ± SEM for 24 rats per group. SEM, standard error of the mean. *P < 0.05 compared with the rats on adenine-free diet; ^†^ *P* < 0.05 compared with adenine-vehicle group; ^‡^ *P* < 0.05 compared with adenine-paricalcitol group

Serum creatinine or BUN levels were similar among the four groups before initiation of the adenine diet (Fig. 1a, b). Two weeks after the adenine diet, the three groups of rats on the adenine diet had significantly increased serum creatinine and BUN compared with the rats on the adenine-free diet, indicating uremia and decreased kidney function induced by the adenine diet. The increases in serum creatinine and BUN progressed over time in the rats on the adenine diet. At 4 weeks, the three groups of rats on the adenine diet had further increases in serum creatinine and BUN compared with the respective parameters at week 2. No significant difference in serum creatinine or BUN was observed between vehicle- and paricalcitol-treated rats. Serum creatinine and BUN were significantly lower in etelcalcetide-treated rats compared with vehicle- or paricalcitol-treated rats.

### Plasma PTH and Serum FGF23, Calcium, and Phosphorus

**Fig. 2:**
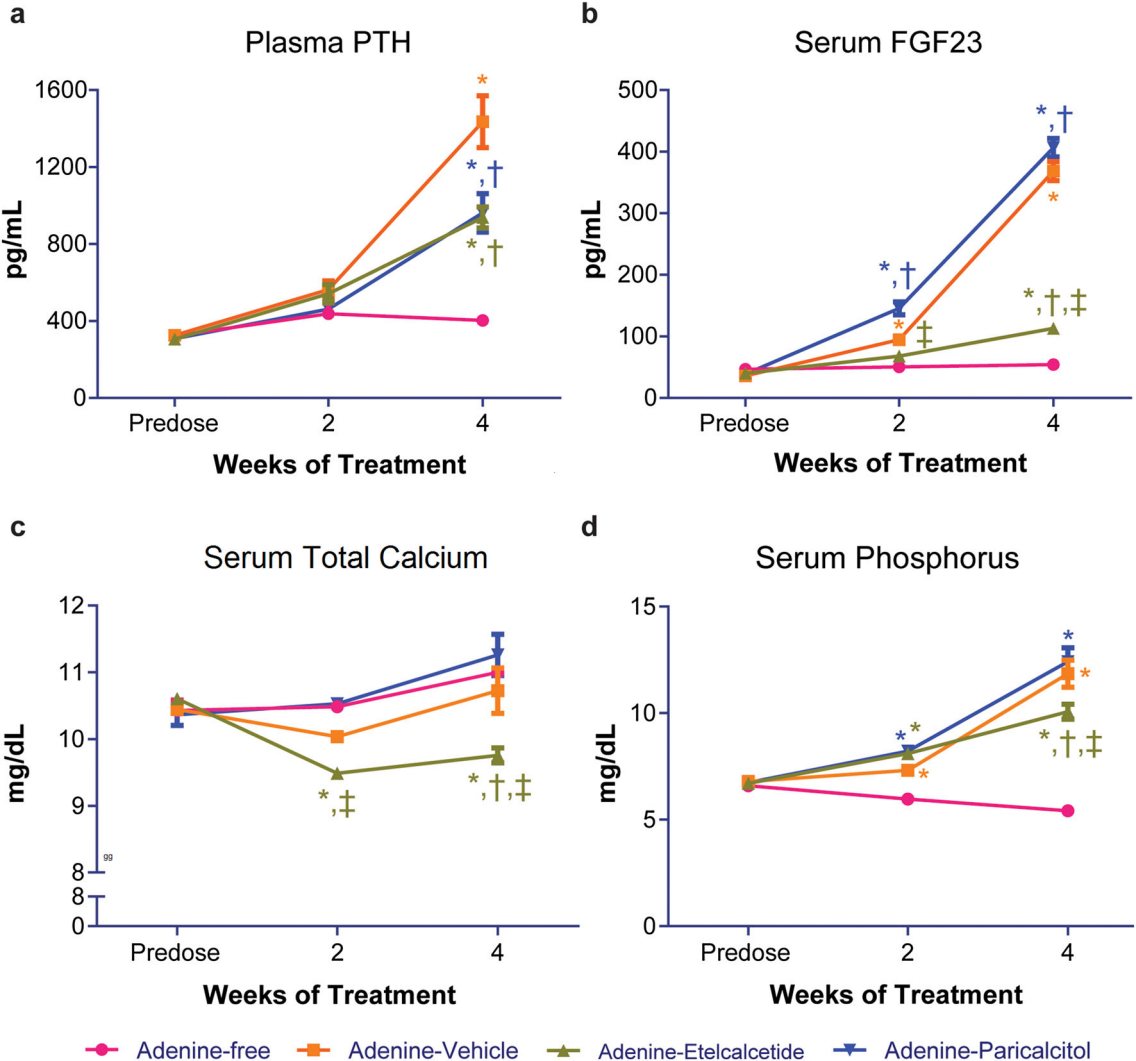
Plasma PTH, serum FGF23, total calcium, and phosphorus before and after treatment in rats on adenine or adenine-free diet. **a** Plasma PTH concentration. **b** Serum FGF23. **c** Serum total calcium concentration. **d** Serum phosphorus concentration. Data are expressed as mean ± SEM for 24 rats per group. PTH, parathyroid hormone; SEM, standard error of the mean. *P < 0.05 compared with the rats on adenine-free diet; ^†^ *P* < 0.05 compared with adenine-vehicle group; ^‡^ *P* < 0.05 compared with adenine-paricalcitol group

Consistent with uremia induced by the adenine diet, plasma PTH increased significantly in all three groups of uremic rats compared with nonuremic controls at week 4 (Fig. 2a; Table 2). Plasma PTH was significantly lower in etelcalcetide- and paricalcitol-treated uremic rats compared with vehicle-treated uremic rats at week 4. Similar results were observed when serum was used for PTH measurements (data not shown). Serum FGF23 increased significantly in all three groups of uremic rats compared with nonuremic rats at week 4 (Fig. 2b; Table 2), with the greatest values observed in the paricalcitol-treated uremic rats and the lowest values observed in etelcalcetide-treated uremic rats. Serum FGF23 was significantly greater in paricalcitol-treated uremic rats at both weeks 2 and 4 compared with vehicle-treated uremic rats. In contrast, serum FGF23 was significantly lower in etelcalcetide-treated uremic rats at week 4 compared with vehicle- or paricalcitol-treated uremic rats. Serum total calcium slightly increased in paricalcitol-treated uremic rats compared with vehicle-treated uremic rats (Fig. 2c; Table 3). In contrast, serum total calcium was significantly lower in etelcalcetide-treated uremic rats compared with vehicle- or paricalcitol-treated uremic rats at week 4. Serum phosphorus increased significantly at weeks 2 and 4 in all three groups of uremic rats, with the greatest increase observed in vehicle- or paricalcitol-treated uremic rats, and the lowest increase observed in etelcalcetide-treated uremic rats at week 4 (Fig. 2d; Table 3). Serum phosphorus was significantly lower at week 4 in etelcalcetide-treated uremic rats compared with vehicle- or paricalcitol-treated uremic rats.

**Table 2:** Comparative effects of etelcalcetide versus paricalcitol on plasma PTH and serum FGF23 in a rat model of uremia induced by dietary adenine

Diet	Treatment	Plasma PTH (pg/mL)			Serum FGF23 (pg/mL)		
		Predose	Week 2	Week 4	Predose	Week 2	Week 4
Adenine-free	Vehicle (*n* = 24)	323 ± 24	438 ± 56	404 ± 26	47 ± 1	51 ± 1	55 ± 2
Adenine	Vehicle (*n* = 24)	326 ± 24	563 ± 46	1435 ± 135^a^	36 ± 1	95 ± 7^a^	368 ± 16^a^
Adenine	Etelcalcetide (*n* = 24)	306 ± 21	541 ± 48	938 ± 55^a,b^	40 ± 2	68 ± 4^c^	113 ± 8^a,b,c^
Adenine	Paricalcitol (*n* = 24)	309 ± 18	462 ± 30	962 ± 101^a,b^	39 ± 2	146 ± 11^a,b^	407 ± 15^a,b^

Results are expressed as mean ± standard error of mean (SEM) for 24 rats per group

^a^ *P* < 0.05 versus vehicle-treated rats without adenine diet

^b^ *P* < 0.05 versus vehicle-treated rats fed with adenine diet

^c^ *P* < 0.05 versus paricalcitol-treated rats fed with adenine diet

**Table 3:** Comparative effects of etelcalcetide versus paricalcitol on serum calcium and phosphorus in a rat model of uremia induced by dietary adenine

Diet	Treatment	Serum calcium (mg/dL)			Serum phosphorus (mg/dL)		
		Predose	Week 2	Week 4	Predose	Week 2	Week 4
Adenine-free	Vehicle (*n* = 24)	10.4 ± 0.2	10.5 ± 0.1	11.0 ± 0.1	6.6 ± 0.1	6.0 ± 0.1	5.4 ± 0.1
Adenine	Vehicle (*n* = 24)	10.4 ± 0.1	10.0 ± 0.1	10.7 ± 0.3	6.8 ± 0.1	7.3 ± 0.2^a^	11.9 ± 0.6^a^
Adenine	Etelcalcetide (*n* = 24)	10.6 ± 0.1	9.5 ± 0.1^a,c^	9.8 ± 0.1^a,b,c^	6.7 ± 0.1	8.1 ± 0.2^a^	10.1 ± 0.3^a,b,c^
Adenine	Paricalcitol (*n* = 24)	10.4 ± 0.2	10.5 ± 0.1	11.3 ± 0.3	6.7 ± 0.2	8.2 ± 0.3^a^	12.4 ± 0.7^a^

Results are expressed as mean ± standard error of mean (SEM) for 24 rats per group

^a^ *P* < 0.05 versus vehicle-treated rats without adenine diet

^b^ *P* < 0.05 versus vehicle-treated rats fed with adenine diet

^c^ *P* < 0.05 versus paricalcitol-treated rats fed with adenine diet

### Kidney Histology

**Fig. 3:**
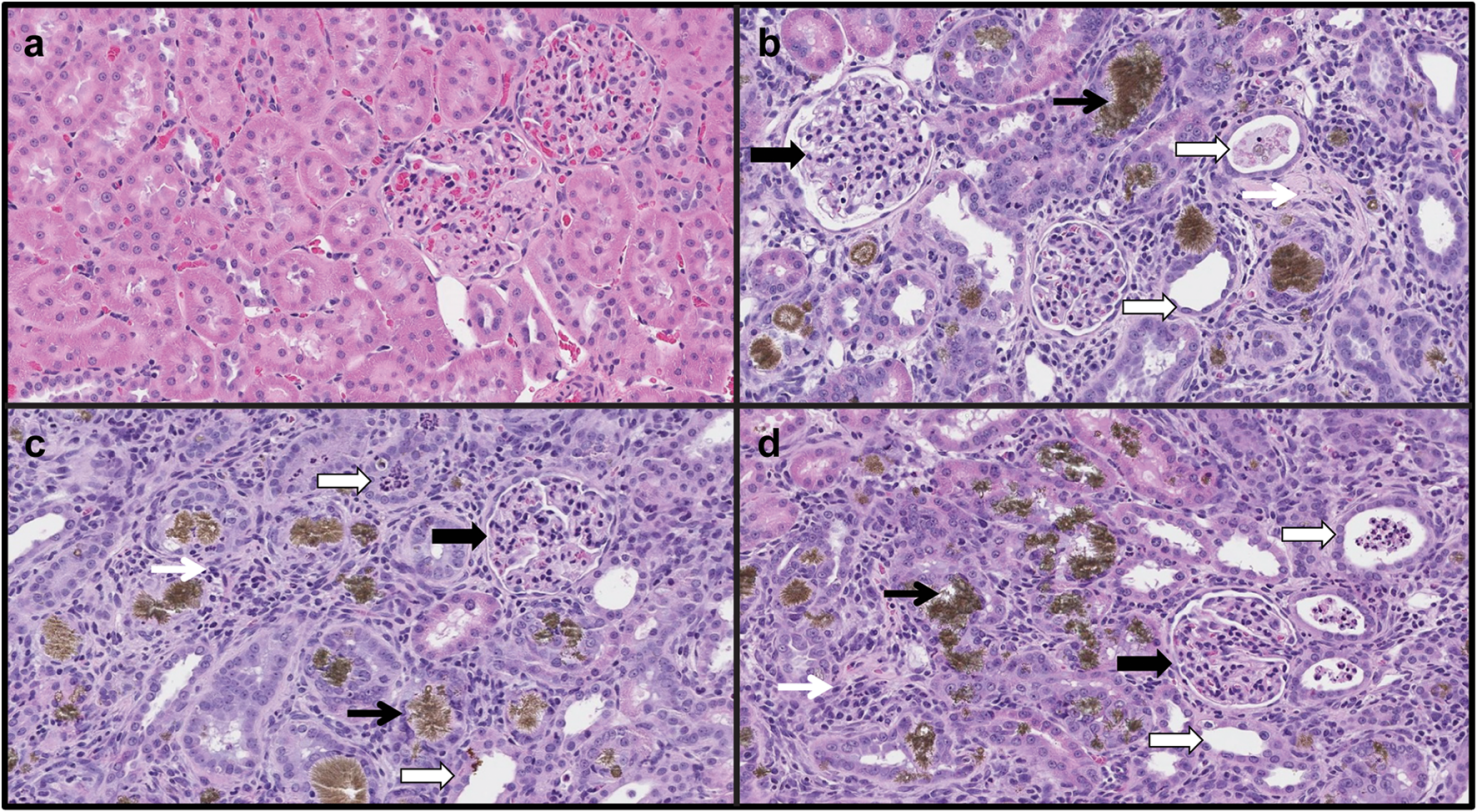
Representative photomicrographs of kidney cortex sections after hematoxylin (H) and eosin (E) staining (H&E, ×200) under light microscope. **a** Rat on adenine-free diet, showing normal kidney architecture and histology, H&E. **b** Vehicle-treated rat on adenine diet. **c** Etelcalcetide-treated rat on adenine diet. **d** Paricalcitol-treated rat on adenine diet. Thick black arrows indicate hypercellular multilobulated glomeruli. Thin black arrows indicate intratubular crystal formation. Thick white arrows indicate intratubular cellular debris and dilated tubules with attenuated epithelium. Thin white arrows indicate interstitial expansion and inflammatory cells infiltration. All rats on adenine diet showed a similar severity of diffuse renal tubular injury

Similar incidence and severity of moderate-to-marked (grade 4–5) diffuse tubulointerstitial injury of entire renal cortex were present in all rats on adenine diet (Fig. 3; Table 4). These findings were characterized by tubular degeneration, necrosis, and dilation, with epithelial attenuation, intratubular cellular debris, and crystal deposition. The intersitium was diffusely expanded by mixed inflammatory cells and fibroblasts (Fig. 3). Etelcalcetide or paricalcitol administrations did not ameliorate incidence or severity of renal injury.

**Table 4:** Histologic scoring of tubulointerstitial injury in kidneys

	Adenine-free (*n* = 24)		Adenine-vehicle (*n* = 24)	Adenine-etelcalcetide (*n* = 24)	Adenine-paricalcitol (*n* = 24)
Rats with grade 4, *n* (%)		0	7 (29%)	6 (25%)	7 (29%)
Rats with grade 5, *n* (%)		0	17 (71%)	18 (75%)	17 (71%)
Average histologic score (mean ± SEM)		0	4.71 ± 0.09*	4.75 ± 0.09*	4.71 ± 0.09*

Semiquantitative histologic scoring was assigned a grade of 0–5 according to the extent of damaged tubulointerstitial area in the renal cortex: 0, normal; grade 1, <10%; grade 2, 10–25%; grade 3, 25–50%; grade 4, 50–75%; and grade 5, 75–100%. No histologic abnormalities of any severity were present in the adenine-free group. Numbers in parenthesis indicate the percentage of rats with grade 4 or 5. SEM, standard error of the mean

*P < 0.05 versus rats on adenine-free diet

### Parathyroid Hyperplasia and Proliferation

**Fig. 4:**
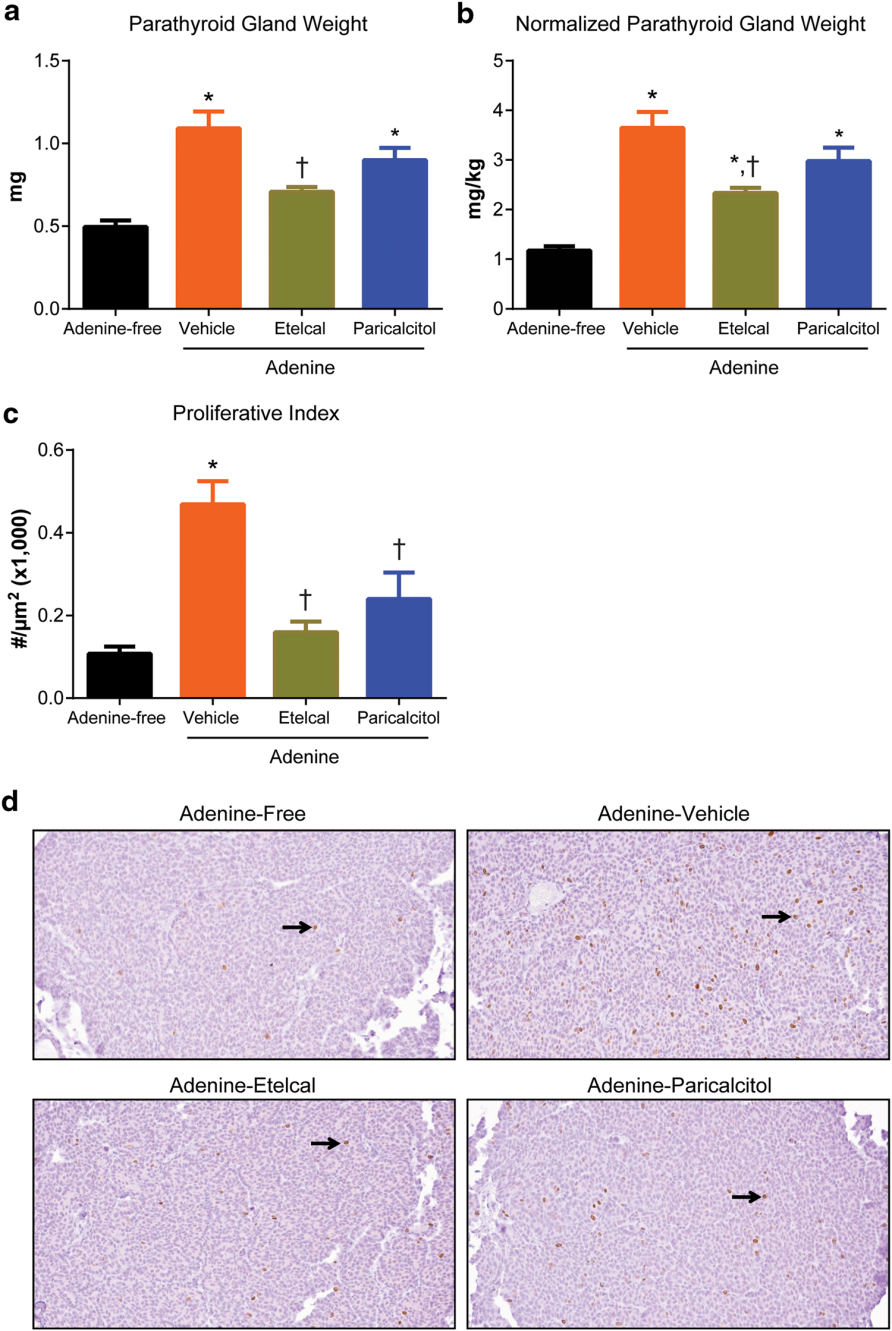
Parathyroid hyperplasia and proliferation index after treatment in rats on adenine or adenine-free diet. **a** Parathyroid gland weight. **b** Normalized parathyroid gland weight (parathyroid gland weight/body weight). **c** Parathyroid proliferative index (Ki-67-stained parathyroid chief cell numbers/tissue area). **d** Representative photomicrographs of parathyroid sections after Ki-67 IHC staining (×200) under light microscope. Dark brown nuclei indicate Ki-67-stained parathyroid chief cells. Data are expressed as mean ± SEM for 16–20 rats per group. Etelcal, etelcalcetide; IHC, immunohistochemistry; SEM, standard error of the mean. *P < 0.05 compared with the rats on adenine-free diet; ^†^ *P* < 0.05 compared with adenine-vehicle group; ^‡^ *P* < 0.05 compared with adenine-paricalcitol group

Parathyroid gland weight was significantly greater in vehicle-treated uremic rats than nonuremic rats at the end of study (Fig. 4a). Parathyroid gland weight was significantly lower in etelcalcetide-treated uremic rats than vehicle-treated uremic rats and was not statistically different from that in nonuremic controls. Parathyroid gland weight in paricalcitol-treated uremic rats did not differ significantly from vehicle-treated uremic rats. Similar results were observed when parathyroid gland weight was normalized to body weight (Fig. 4b). To study parathyroid chief cell proliferation, the parathyroid tissue was stained with Ki-67, a marker of cell proliferation. Parathyroid proliferative index, defined as area-normalized Ki-67-positive cell number, was 4.4-fold greater in vehicle-treated uremic rats than nonuremic rats at the end of study (Fig. 4c, d). In contrast, parathyroid proliferative index was significantly lower in etelcalcetide- or paricalcitol-treated uremic rats than vehicle-treated uremic rats and was not statistically different from the nonuremic rats.

### Aortic Calcification

**Fig. 5:**
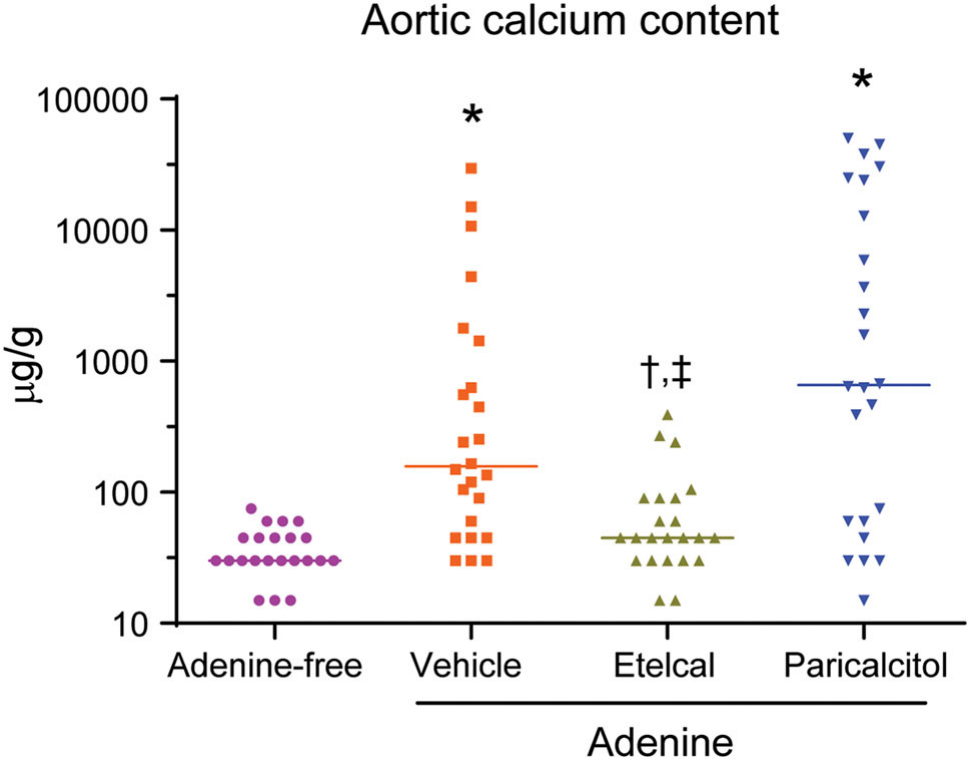
Aortic calcium content. Data represent individual rats with median value, with *n* = 23–24 per group. Etelcal, etelcalcetide. *P < 0.05 compared with the rats on adenine-free diet; ^†^ *P* < 0.05 compared with adenine-vehicle group; ^‡^ *P* < 0.05 compared with adenine-paricalcitol group. *Y*-axis was log-scaled for better visualization of individual rat with lower calcium profile

Aortic calcium content, an indicator of calcification, was significantly greater in vehicle- or paricalcitol-treated uremic rats than nonuremic rats (Fig. 5). Aortic calcium content was significantly lower in etelcalcetide-treated uremic rats (median = 45 µg/g) than in vehicle-treated uremic rats (median = 158 µg/g) and was not statistically different from that in nonuremic rats (median = 30 µg/g). Nine and 15 out of 24 rats (38 and 63%) had calcium content greater than 400 µg/g in vehicle- and paricalcitol-treated uremic rats, corresponding to median values of 158 and 660 µg/g, respectively. In contrast, no etelcalcetide-treated uremic rats had calcium content greater than 400 µg/g. A comparison in aortic calcium content between paricalcitol- and vehicle-treated uremic rats demonstrated a *P* value of 0.077.

### Aortic Histology

**Fig. 6:**
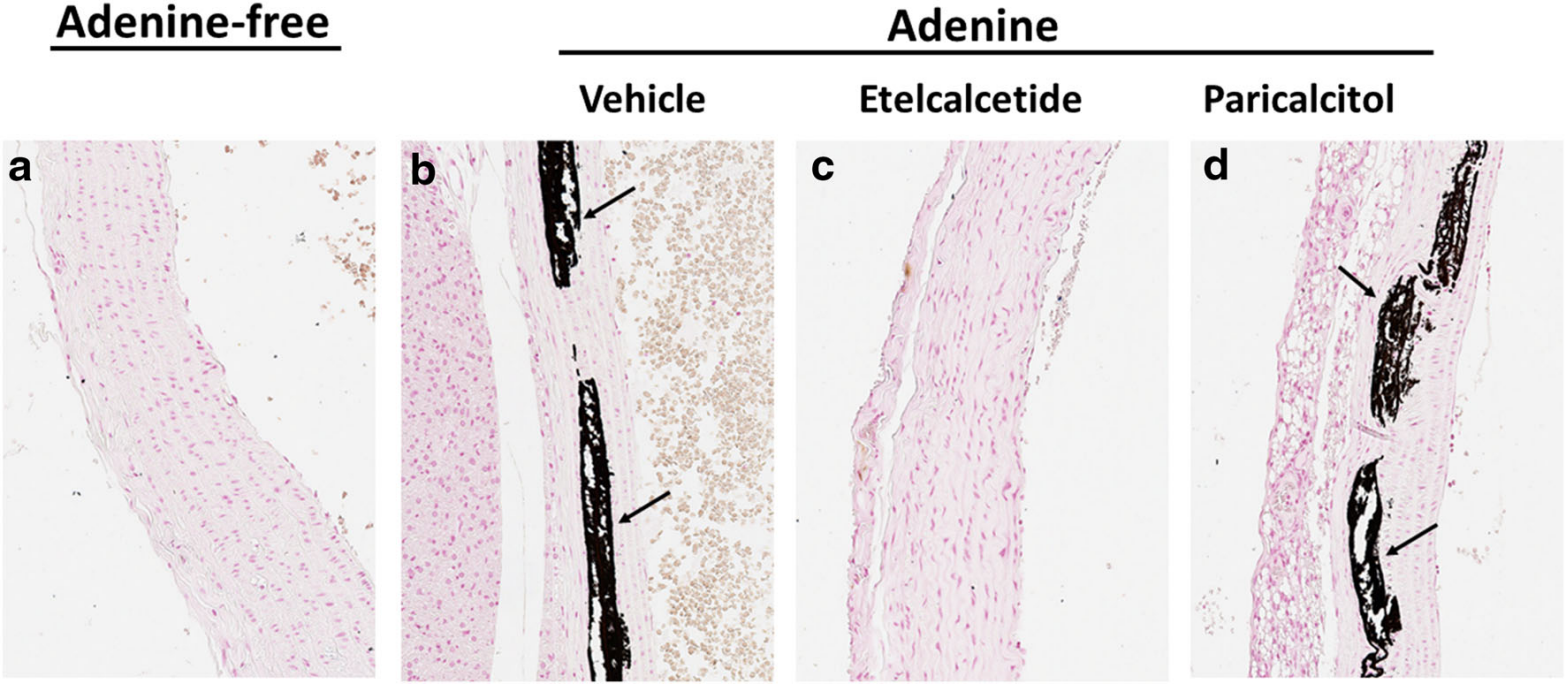
Representative photomicrographs of aortic tissue with von Kossa staining. Control rat aorta shows normal vascular structural arrangement lacking calcification (**a**). Vascular calcification in medial layer vascular smooth muscle cells was evident in adenine-vehicle (**b**), but not in the etelcalcetide-treated group (**c**). Vascular calcification was prominent in paricalcitol-treated groups (**d**). Longitudinal aortic sections were stained with von Kossa for the identification of calcification. *Black arrows* indicate medial calcification. All magnification is ×200

A similar incidence and severity of aortic calcification was present in the aorta in vehicle- and paricalcitol-treated uremic rats (Table 5; Fig. 6). No observable calcification was observed in longitudinal specimens of control nonuremic or etelcalcetide-treated uremic rats.

**Table 5:** Incidence and severity of histologic aortic mineralization

Animals examined	Adenine-free (*n* = 24)	Adenine diet		
		Vehicle (*n* = 24)	Etelcalcetide (*n* = 23)	Paricalcitol (*n* = 24)
No mineralization	24	15	23	13
Minimum	0	1	0	0
Mild	0	2	0	1
Moderate	0	0	0	2
Marked	0	6	0	8
Total incidence	0	9/24 (38%)	0	11/24 (46%)

Semiquantitative histologic scoring of aortic wall mineralization: minimal = focal patchy vascular mineralization of tunica media detectible, mild = up to 10% of total specimen mineralized, moderate = 10–50% of specimen mineralized, Marked >50% of specimen mineralized

## Discussion

In the adenine model of SHPT and vascular calcification, etelcalcetide or low-dose paricalcitol administrations resulted in similar reductions in plasma PTH and parathyroid proliferation index. The PTH reduction was about 33% in both etelcalcetide- or paricalcitol-treated uremic rats compared with vehicle-treated uremic rats at week 4. However, etelcalcetide and paricalcitol had different effects on serum total calcium, phosphorus, FGF23, and vascular calcification. Importantly, 38 and 63% of rats had aortic calcium content greater than 400 µg/g in vehicle- or paricalcitol-treated uremic rats, respectively, while no etelcalcetide-treated rats had aortic calcium content greater than 400 µg/g. Histological evidence of aortic calcification (von Kossa staining) was evident in vehicle- or paricalcitol-treated uremic rats, but not in etelcalcetide-treated rats nor control (nonuremic) rats. The results demonstrated fundamental differences between etelcalcetide and paricalcitol in the control of mineral homeostasis and vascular calcification in this preclinical model. The observed effects of etelcalcetide and paricalcitol on circulating PTH, calcium, phosphorus, and FGF23 in this study are in agreement with the results observed in clinical studies with etelcalcetide or cinacalcet [5, 9, 24], and paricalcitol [8, 10].

Our results provide new insight into the effects of etelcalcetide on vascular calcification and are in line with previous results obtained in clinical studies with cinacalcet [3, 4] or the use of cinacalcet and calcimimetic compounds in preclinical models of SHPT and vascular calcification [11, 12]. The data herein also confirm the strong association of elevated calcium and phosphate to the initiation and progression of vascular calcification in CKD. In this study, etelcalcetide decreased plasma PTH, serum total calcium, and phosphorus, and attenuated the development of vascular calcification. In contrast, low-dose paricalcitol decreased PTH similarly but had no significant effects on serum total calcium and phosphorus, and did not attenuate the development of vascular calcification. In addition, etelcalcetide effects on vascular calcification may derive from its direct effects on vascular calcification. Other calcimimetics have also been shown to have a direct effect on vascular cells [30]. CaSR expression has been demonstrated in endothelial cells  [31] and vascular smooth muscle cells (VSMC) [32] and calcimimetic direct activation of the CaSR has been shown to increase matrix Gla protein (calcification inhibitor) expression in the arterial wall, both in in vivo and in vitro [33], [14], [34]. It is plausible that etelcalcetide may have similar direct effects on vascular calcification.

Although calcitriol has been shown to increase vascular calcification in both hemodialysis patients and preclinical studies [6, 7, 16], the effects of paricalcitol on vascular calcification remain controversial. In an effort to further understand the pharmacologic effects of paricalcitol on vascular calcification, 42 ng/kg was selected in our study. This dose was selected based on its nonsignificant effects on serum calcium [35], in order to avoid the contribution of hypercalcemia induced by higher doses of paricalcitol to the development of vascular calcification. Indeed, this dose of paricalcitol reduced PTH and did not significantly increase serum calcium, but was associated with a nominal increase in the percentage of rats with vascular calcification (*P* = 0.077 vs. Adenine-Vehicle). These results support other studies related to the effect of paricalcitol on vascular calcification [18, 19] and confirm the lack of protective effects of paricalcitol on vascular calcification in animal models of SHPT. However, these results do not agree with two previous studies conducted in mice [20, 21] for reasons not well understood. It is important to note that both calcitriol and paricalcitol were shown to reduce vascular calcification in these two mouse studies. As suggested in a recent editorial [36], the inhibitory effect of calcitriol or paricalcitol on vascular calcification in mouse studies should be interpreted with caution. The administration of calcitriol or paricalcitol resulted in reduction in serum phosphorus in the aforementioned studies, which is not usually observed in CKD patients treated with calcitriol or paricalcitol [10].

Increased serum levels of the phosphaturic hormone FGF23 have been associated with progression of CKD [37], vascular calcification [38, 39], left ventricular hypertrophy [40], and cardiovascular mortality [41]; however, the impact of FGF23 on vascular calcification remains controversial [42]. In addition, FGF23 has been shown to have a direct effect on myocardium [43, 44]. In rats fed adenine diet, FGF23 was significantly lower in etelcalcetide-treated rats, but significantly higher in paricalcitol-treated rats compared with vehicle-treated rats. FGF23 reduction was also reported in clinical studies with cinacalcet or etelcalcetide in hemodialysis patients [5, 9, 24]. In phase I clinical studies, a single administration of etelcalcetide dose-dependently decreased FGF23 in hemodialysis patients and healthy male subjects [24, 45]. The calcimimetic treatment effect on FGF23 is consistent with emerging data suggesting that PTH can directly stimulate FGF23 production [46] and is in line with FGF23 regulation by serum calcium, phosphorus, and their interactions [47, 48]. It is interesting to note that increased vascular calcification and increased phosphorus levels were observed [49] in preclinical studies using an FGF23 antibody to reduce FGF23 levels in a different model of CKD. Therefore, the ability of etelcalcetide to better maintain mineral homeostasis, while decreasing FGF23 is crucial for the prevention of vascular calcification in this model and may suggest a safe way of lowering FGF23.

The observation that FGF23 levels remain elevated despite lower PTH levels after 4 weeks of paricalcitol treatment suggests that this vitamin D analog may have a PTH-independent effect to regulate FGF23 synthesis/production. A single injection of 1, 25 dihydroxyvitamin D_3_ increased FGF23 mRNA in bone and increased serum FGF23 concentration in mice. Analysis of the FGF23 gene promoter revealed the presence of a vitamin D response element [50]. In contrast, specific deletion of vitamin D receptor in bone cells decreases FGF23 production [51]. Furthermore, increased serum FGF23 was observed in hemodialysis patients treated with vitamin D and its analog including alfacalcidol and paricalcitol [8, 9].

Interestingly, parathyroid cell proliferation, as assessed by Ki-67 marker, was significantly reduced by both etelcalcetide and paricalcitol, whereas parathyroid gland weight was only reduced in the etelcalcetide-treated animals. The results suggest that Ki-67 may be a more sensitive measure than the parathyroid gland weight. Indeed, the difference in the parathyroid gland weight between adenine and control groups was about 2-fold but the difference in area-adjusted Ki-67-positive cells is about 5-fold.

There are limitations to the studies presented in this article. First, biomarkers of renal injury were increased to a lesser degree in etelcalcetide-treated, uremic rats than in vehicle-treated uremic rats, suggesting that renal function was better preserved upon etelcalcetide treatment. The reason for this improvement is not well understood, since kidney histology showed a similar degree of tubulointerstitial injury in vehicle and etelcalcetide- or paricalcitol-treated rats. Renal protective effects have been reported with calcimimetics in other preclinical studies [52, 53]. Future studies measuring glomerular filtration rate would provide a better indication of differences in renal function between treatment groups. Nevertheless, better kidney function in the etelcalcetide group may have contributed, at least in part, to the differences in vascular calcification between the etelcalcetide- and paricalcitol-treated groups. It is important to note that impairment in kidney function, as reflected by increased serum creatinine and BUN, was similar between etelcalcetide- and paricalcitol-treated rats following 2 weeks of treatment. Under this condition, FGF23 was significantly higher in paricalcitol group than etelcalcetide group, indicating a fundamental difference in FGF23 regulation between etelcalcetide and paricalcitol. Second, the incidence of vascular calcification was relatively low in our study. Only 38% of rats developed vascular calcification in vehicle-treated SHPT group, despite the fact that we used the same low protein with adenine diet to increase the incidence of vascular calcification as reported previously [29]. To overcome the relatively low incidence of vascular calcification, large animal numbers per group were used in our study. Third, serum calcium is measured only at 24 h post paricalcitol dosing. It is not known whether paricalcitol caused transient hypercalcemia at earlier time points, which may have contributed to the development of vascular calcification in this study. Fourth, while the body weight loss was consistent among three adenine groups, adenine diet results in a 25% drop in body weight which may introduce confounding metabolic factors. Under this condition, no evident cardiac hypertrophy was detected in adenine group (data not shown), limiting our ability to study the consequence of vascular calcification on cardiac hypertrophy in the same study.

In summary, these studies demonstrated that etelcalcetide attenuated the progression of SHPT, lowered FGF23, and prevented vascular calcification in a rat model of CKD induced by an adenine diet. Low-dose paricalcitol similarly attenuated the progression of SHPT but raised FGF23 and did not prevent the development of vascular calcification in the same model.
